# Parents' Experiences of Caring Responsibility for Their Adult Child with Schizophrenia

**DOI:** 10.1155/2016/1958198

**Published:** 2016-02-04

**Authors:** Ann Blomgren Mannerheim, Ulla Hellström Muhli, Eleni Siouta

**Affiliations:** ^1^Karolinska Institute, Division of Nursing, Department of Neurobiology, Care Science and Society, 171 77 Stockholm, Sweden; ^2^Department of Sociology, Uppsala University, Box 624, 751 26 Uppsala, Sweden; ^3^Department of Learning, Informatics, Management and Ethics (LIME), Karolinska Institute, 171 77 Stockholm, Sweden; ^4^Sophiahemmet University, Box 5605, 114 86 Stockholm, Sweden

## Abstract

As a consequence of the latest psychiatry-related reform in Sweden and its implementation, relatives and family members have taken over from the formal healthcare system significant responsibility for the care of persons with a mental disability and illness. The aim of this study was to systematically describe and analyze the experiences of parents' informal care responsibility. The questions were, what are the experiences around parents' informal care activities and responsibilities and how do parents construct and manage their caring responsibility and with what consequences? Semistructured in-depth interviews were conducted (16 hours of recorded material) with eight parents who were all members of the Interest Association for Schizophrenia (*Intresseföreningen för Schizofreni* (IFS)) in Sweden. A mixed hermeneutic deductive and inductive method was used for the interpretation of the material. The parents endow their informal caring responsibility with meaning of* being a good*,* responsible*, and* accountable* parent with respect to their social context and social relationships as well as with respect to the psychiatric care representatives. In this tense situation, parents compromise between elements of struggle, cooperation, avoidance, and adaption in their interaction with the world outside, meaning the world beyond the care provision for their child, as well as with the world inside themselves.

## 1. Introduction

This study focuses on parents' experiences of informal care responsibility in relation to the responsibility for taking care of their adult child with a mental illness.

The term mental illness is often used as a collective term for less serious mental disorders such as anxiety. However, the term herein refers to severe mental illness that is expressed in a syndrome that can be verified on the basis of different criteria for psychiatric diagnosis [1].

Psychiatric care in Sweden has undergone several organizational changes after the mental health reform of 1995 [2]. Concretely, the changes were mainly decommissioning of large psychiatric institutions with a significant reduction in the number of inpatient care facilities in favor of the expansion of psychiatric outpatient care. This reform, from a policy perspective, meant that outpatient activities related to support and service, housing, employment, leisure, and the social needs of the mentally ill individuals were transferred from the county councils to the municipalities [3]. Thus, the reform was not a reform of specialized psychiatry but was instead an attempt to improve the lives of the mentally disabled and the sick in the community [4]. However, problems arose quickly since no sector entirely managed the needs that existed within the framework of the current outpatient care [3, 5–7]. A significant responsibility for care has also been transferred from the formal psychiatric care to related parties, that is, the families of the mentally ill individual. The definition of formal care here refers to “the healthcare organization,” “healthcare professionals,” “psychiatric institutions,” “psychiatric care givers,” and “formal care givers.”

Under the policy framework described above, relatives constitute an important source of support for the care of individuals with mental illness. The family's informal care responsibility has been shown to have positive effects on the sick individual's rehabilitation, as well as on the patient's daily life, especially since relatives offer continuity in the care based on their knowledge of the sick individual [8–10].

However, at the individual level, the policy-formulation means that the parents of the adult child with schizophrenia (ACWS) have increased responsibilities for the care of their sick adult child, given that the child will now be treated either at the parental home or in some form of group housing [11]. The interdisciplinary field of caring studies has recently produced research showing that in many cases this responsibility represents an unacceptable burden on the family including social stigma, refusal of treatment, and stress [12–14]. The informal caregiving role puts a burden on the parents but also leads to economic problems, limitations in the parent's social life, and emotional problems [15–18]. Karanci [19] stresses increased incidences of family conflicts, divorces, and siblings who are maltreated. It has been shown that these care responsibilities affect the physical and mental health of the family member [15].

There have also been an increasing number of studies of the* welfare and mental health reform* in Sweden focused on the methods of planning and implementing the reform, the target group of the reform, and resulting deinstitutionalization [11, 20–24]. However, only a few studies in Sweden focus on parents' experiences and their perspectives on informal care responsibility for their ACWS. There is also a lack of knowledge about the formal psychiatric care support available for relatives taking care of the individuals with a mental illness [4, 10, 16, 25] and in particular support for relatives' own well-being [26, 27].

It is against this background that we framed our paper analyzing parents' experiences of their informal care responsibility related to their ACWS.

The questions posed are, what are the experiences around the parents' informal care activities and responsibilities and how do parents construct and manage their caring responsibility and with what consequences?

## 2. The Topic of Informal Care and Care Responsibility

Informal care is taken to mean care contributions made by relatives outside the framework of the public care services provided by state, the county council, or the municipality and in contrast to the formal care that refers to the services and care provided by the public system [12].

Unlike formal care, the specific nature of informal care depends on relatives being “available” practically around the clock [12]. Studies indicate that this constant accessibility is often the reason why relatives perceive informal care as an unacceptable burden [12, 14, 28].

International research has sought to distinguish between* objective* and* subjective* aspects of the burden of informal care. Objective aspects deal with giving up working, wholly or partly to be able to provide care, financial sacrifices, and sacrifices to the caregiver's own social relations and time [12, 15–18]. Subjective aspects refer to the caregivers' emotional and physical reactions to the situation [12].

The* concept of caring* captures the meaning of giving care to those who cannot perform a particular chore. Thereby it also captures the dimensions of a disadvantage in the caring relation. Accordingly, the specific logic of the care (the caring rationality) involves aspects of asymmetries, morality, and human values at the same time as the care must be planned, valued, and acted upon [29]. The objective of care is well-being and quality of life. Parental informal care responsibility to their ACWS can therefore be understood on the basis of this theoretical foundation, to increase the child's well-being and quality of life by being present in the existential as well as spatial and temporal meaning. According to Mayeroff [30], caring is about initiating a person's health and well-being processes by providing room for growth and development. In this study the authors intend to conceptualize this complex interplay of meaning-making, expectations, feelings, and attitudes, interpreted in interviews with parents to ACWS and related to their care responsibility.

Theoretically, the meaning-making can be traced to a social construction perspective when meaning-making of the parental caring responsibility is embedded in cultural meaning and the informal caring responsibility is perceived as parents' social reality and knowledge [31]. Furthermore, the parental caring responsibility is socially constructed at the experiential level, based on how individuals (here parents) come to understand and live with their parental and informal care responsibility. This approach can make a significant contribution to our understanding of the social and experiential dimensions of caring responsibility and it can help us broaden policy deliberations and decisions.

## 3. Methods

The aim of this study is to systematically describe and analyze the meaning of parents' care responsibility for their ACWS. This means that qualitative in-depth interviews were used to elicit parents' experiences and meaning-making, which were analyzed by a hermeneutic mixed deductive and inductive method [32]. This kind of hermeneutic interpretative perspective enables detailed analyses to be undertaken of the meaning of the parents' informal care responsibility as experienced (inductively) within the context of daily life and social interaction with others as well as with representatives of the psychiatric healthcare system. This analysis makes it possible to find interesting proposals for how various phenomena are related, here the parents' meaning-making incorporated in the social context or the everyday culture in which they live. This perspective also makes it possible to analyze parents' understanding of their own performance of self [33] and to analyze the data in regard to the theory of caring rationality [29] (deductively) and for confirming social construction as theoretical framework. This method extends understanding from what is called a hermeneutic circle. However, in this interpretative perspective it is also important to give an account of the researchers' prior understanding that may influence interviews and analysis. In addition, as researchers, our background qualifications in psychiatric care as well as in other care settings (work experience as a specialist nurse in psychiatric care, as a registered nurse in the cardiac care, and as a manager of elderly care) contribute some ethnographic knowledge and preunderstandings of such a context. The understanding of the hermeneutic interpretative perspective toward the social construction perspective of the individual as a meaning-making subject also requires knowledge about the cultural context and knowledge that we possess to varying degrees.

### 3.1. Procedures and Data

The data corpus consists of eight interviews (a total of 16 hours of recorded interview material) with an equal number of parents of mentally disabled and ill persons who are members of an interest association for schizophrenia named (*Intresseföreningen för Schizofreni* (IFS))* the Interest Association for Schizophrenia*, in Sweden. The association is a disability organization that organizes people with schizophrenia and related psychoses, their relatives, and other interested persons. The association works to increase understanding of schizophrenia and related psychotic disorders and the disabilities that the disease brings. The organization consists of about 50 local associations and districts with 3,500 members throughout Sweden [34]. A call for participants in the study went to one local association in the middle of Sweden.

To get participants for the study, the senior manager at the local association for schizophrenia was contacted by telephone and was provided with information about the study. The manager informed members about the study and she advertised for volunteer participants in the study. Eight members reported an interest in participating and represent the study participants. The purpose of the study was first presented orally and then in writing. An information letter describing the purpose of the study and its design was sent to those who expressed an interest in participating.

### 3.2. Participants

The participants—three men and five women between 52 and 63 years old—had all at least five years' experience of informal caring. Four of the eight parents were married at the time of the interviews (although one was in the process of getting divorced) and four lived as single parents. Only three of the eight participants worked full-time. The others were receiving sickness benefits to various degrees. The target group's ACWS consisted of six sons and two daughters aged 22–31 (Table 1).

### 3.3. Ethics and Data Collection

Ethical aspects were emphasized in accordance with the Helsinki Declaration of 1975, as revised in 2008 [35], and with the Swedish Government Proposition [36] and the Governmental Act [37]. The participants were informed that participation in the study was voluntary and that they could at any time cancel participation without giving any reasons. The participants were also assured of confidentiality during the interviews.

The interviews were conducted by the first author (Ann Blomgren Mannerheim). All the interviews took place at the time and place of the participant's choice: at the association's premises, their own workplace, or the participant's home. The interviews maintained a semistructured form, based on an interview guide. The interviews began by gathering general background information about age, working hours (full-time, part-time), and the child's age and gender, followed by main interview questions regarding (i) care responsibility, (ii) life situation, (iii) their own health, and (iv) manifesting confirmatory care from the psychiatric healthcare system. Each interview took about two hours and was conducted in an informal open dialogue, allowing interviewees to answer in many different ways and the interviewer to ask follow-up questions.

The parents interviewed during the research are seen primarily as informers, contributing knowledge of their experiences and of their meaning-making of the informal care responsibility. In our role as researchers, our interest focused on the information rather than the interaction, even though these aspects interlace, as the interview itself is an interactional activity. The interviews were tape-recorded and transcribed verbatim using a written and readable language. This resulted in a reduced level of detail in the transcriptions, but the purpose of this was to create more fluent text and description. The transcription excerpts in this paper have been translated from Swedish to English. Translating the parents' accounts is problematic as interpretation and cultural content within the accounts may be lost which is a limitation that should be considered.

### 3.4. Analysis of Data

Parents' experiences and meaning-making were central to the analysis [38]. The data was analyzed qualitatively by a hermeneutic deductive and inductive method for the interpretation. The analysis was done within the framework of the theory of social construction with emphasis on how meanings of phenomena do not necessarily inhere in the phenomena themselves (informal caring responsibility) but develop through interactions in a social context [31]. However, the analysis focused on how this was understood, what meaning it was given as described by the parents (inductively). This method interprets hermeneutics as the hypothetic-deductive and inductive mixed method used on meaning-carrying material created from the “insider's” view (parents') and how the parents come to understand and live with their informal caring responsibility based on their everyday knowledge [39]. The hypothesis that was set up had been created by an internal image of what one could expect to find in the material.

The hermeneutic approach is a constant movement between a part of a text and the contextual whole that it is part of. This method describes the hermeneutical spiral in the form of circles that touch the authors' understanding, the participants' meaning-making, and meaning of the text [38]. In this study, the movement between a part of the text and the contextual whole was to fit the part and the contextual whole into each other to form a meaningful unit. Using this approach, we (all three authors Ann Blomgren Mannerheim, Ulla Hellström Muhli, and Eleni Siouta) moved between understanding the persons, their actions, and ourselves and our horizon of understanding [32]. The data was read separately by each author. We started our analysis to find* statements and experiences* with focus on (i) the meaning of care responsibility and went on to find (ii) the social organization and strategies of the parents' world and further to find (iii) adaption to the caring responsibility. The final categorization was developed to summarize the results and the objectives as well as to create new descriptions and concepts that explain the main interpretation of the study. The analysis of the data is specified in following stages.

**Stage 1 .**

The transcripts were read thoroughly several times to obtain an overall representation and a comprehensive view of the material.

**Stage 2 .**

The data was then read with a first reflection on various themes related to the interview guide.

**Stage 3 .**

Then the data was read with a second reflection and the reflection was moved to the connection of the theoretical framework. This phase was the interpretative phase and meant to deepen our understanding.

**Stage 4 .**

In this stage, we took the first step towards the organization of the data with the help of a coding process. This coding process involved finding text that by its content was marked with a code. In this case, by color selections using different color pens, the coding continued with sorting of data as a thematic and code-driven grouping of the meaningful units in the data.

**Stage 5 .**

As mentioned above, the final categorization was developed to lead to a summary of the results and the objectives as well as to create new descriptions and concepts which explains the main interpretation of the study.

## 4. Findings

### 4.1. The Meaning of Informal Care Responsibility

In the following text, experiences and meaning-making of the parents' informal care responsibility are specified. The main findings were the parents' experience and that they present themselves as attentive and active parents, and they live with this responsibility by trying to create good social relations outside the home. They support and care for their ACWS in different ways and have good contact with the representatives of the formal psychiatric care. However, the most important meaning for parents seems to be to* experience themselves as good and responsible parents*, who care not only about their ACWS, but also about the whole family. This meaning-making is exemplified by the following extracts:

*I have one task and that is to meet xx (her son) and sit and talk, and then… he has really talked about his situation every time and every week and it feels like I have his trust and then I think it's great that he wants to talk so much. I have a feeling that he also feels good about those talks, even if he talks to others as well; there is something special to talk to the mom who know him best.* (Informant)

*The only thing that means something right now is to find some meaningful activity for her (her daughter), for she has not really had any over the last three, four years. It is vital for her actually, because otherwise I do not think she is able to cope with…. *(Informant)

The parents take on their care responsibilities as a challenge and the paid work (employment) outside the informal care responsibility seems as stimulation or allows for “breathing,” as one informant metaphorically put it. This challenge is expressed in the following extract:

*I think I feel good, I feel myself quite happy, sleep well at night even though I have a big workload, now I have a full-time job, plus I take care of my dad who is 90 years plus I have xx (her son) to think about and I'm single, so it is much that lies on me. *(Informant)

Overall, the key issue here is that parents endow their informal caring responsibility with* meaning of being responsible and accountable* within the context of their personal and social relationships. Thus, the analysis shows that the importance of being a good parent with skills to care for his/her adult son or daughter means not only to be a capable parent to the child, but also to show the community and the formal mental health services that the parent takes his/her care responsibilities seriously.  Table 2 summarizes the parents' meaning of their informal care responsibility in theme, categories, and subcategories.

### 4.2. The Social Organization of the Parent's World as Informal Carers in Form of Strategy Projects

The results from the analysis of strategies used for living with the informal care responsibility show four main categories: strategies such as (1)* parental projects*, (2)* work related projects*, (3)* recreational projects*, and (4)* repertoires of controlling the life situation*, on the one hand, and* feelings of being out of control*, on the other.

*The parental project* as a strategy was to be a good parent who felt responsible for their adult children. This was important even if it meant for some that they could not keep up with other works. Parents tried in various ways to support their children, for example, by teaching about finances, by encouraging independence, by offering companionship, and by finding employment.

The parent's own health as well as the child's health is also of importance in the parental project as strategy. The analysis shows that the parents felt that they could relax more and feel better if their son or daughter was in a stable period of the disease process.

Other important health issues were to support the ACWS to follow the treatment plan with proposed activities and to get the child to feel commitment to the family. Another strategy was to support a daughter with companionship, and another strategy was to get the child to follow daily routines. Some of the parents' strategies were to fight for their child so that he/she could receive medical treatment. However, this took up all their time and energy and even contributed to thoughts of giving up life, as exemplified below:

*The strength to fight…but it's not always that I have the strength to do that, sometimes I fall down and then I'll lie down for a couple of days and then I'll get up again …now have they fucking done that again, now damn, I say to myself, and then I start over again, and then I fall down again, and sometimes I feel that I give up – now I'll take all my sleeping pills, now to hell with this, but I don't, but I feel like that sometimes.* (Informant)

In regard to the parental project strategy, results clearly show that parents are looking for various solutions to realize their goals and, on the whole, for existence that one is satisfied with based on the needs that may arise as a result of the child's illness. One important strategy was to uphold the relations within the family, between spouses, or with the child. Parents also report that they receive valuable knowledge about their own self and about their own abilities to realize their life through self-therapy or by consulting people and through psychiatric expertise about the disease affecting their child. The motivation for these strategies seems to be a greater readiness in their parenting, care responsibility, and understanding of their child's illness. In a constant search for knowledge and understanding of mental health and diseases, the parents test and process their own attitudes toward the illness as well as the general view in the society against persons with mental disabilities and illness. Alleviation of difficulties in their lives is also experienced by talking openly to others about their child being mentally ill.

*The work related project* objective was to use paid work as a source of internal development, stimulation, and appreciation. Thus it was an important strategy in their lives and not a burden in addition to the informal care responsibilities. Those parents who particularly emphasized the work as being challenging and stimulating also worked proportionally more, engaged in full-time service, and felt that they had a job that gave them the challenge, stimulation, and opportunities in life. This is expressed as follows:

*No, the work is no problem, I've got a job after all; my boss jokes that now they have paid so much to me, so now I have to stay and work until I'm 67 years old, so from that aspect I am not concerned; that is really nice because it is really important.* (Informant)

In regard to the work and* recreational project* strategies, results show that* work and leisure time* are important assets and strategies in parenting and in the informal care responsibility. Parents stressed the importance of having a job that is stimulating. However, the job also allows space for breathing, which was expressed as follows:

*Work became something to lean against; I simply fled to work, it was important for me to be able to go to work every day because then I switched off everything else.* (Informant)

As shown, work gives the opportunity to break with the everyday caring responsibility and creates contrasts between the role of parent and professional. Having a job cultivates a healthy distance with the informal caring responsibility helping to satisfy parents' own personal needs in the context of informal caring responsibility.

Another strategy was trying to* adapt their working conditions to the current life situation* by reducing the time at work in order to perform their caring responsibilities for their adult child. However, despite this change of life, work remains important and is explained as follows:

*Then, when this clinical picture came of my son, I thought I could find some calmer work. I tried to adapt myself so I only needed to work three days a week. In that way, I had more time over for my son because he was still at home. *(Informant)

When it comes to* the recreational project*, the strategy is to pursue various hobbies or recreational activities. Such activities could be to walk in the mountains, bike, dance, or work with a holiday cottage or organizational activities. These parents have a variety of recreational activities that they describe as important, and such one is shown below:

*We usually hike in the mountains a lot when we're up at the cottage, but this year we did not so much; mostly, there was berry picking and painting…well a little bit of hiking too.* (Informant)

The parents feel that having time to engage in diverse individual activities, from various forms of exercise to travel, is a valuable strategy to realize their own life in the caring responsibility. Social contact with openness about the disease with family, friends, and wider community is another strategy that also offers relief from shame and guilt.

Visiting the Interest Association for Schizophrenia is also viewed as a significant aspect of support for the parents' life circumstances. By joining the association, parents come into contact with other parents in the same situation, which is perceived as supportive of their existence. Parents also felt that they got knowledge and support through advice and lectures given by the association.

As shown, maintaining their social relationships and finding space and time for their own needs were emphasized by the parents as an important strategy to realize their life situation and circumstance and are expressed according to the following:

*I've got my own home that I can go to, and I can meet my friends at my place. I even try to have some fun stuff so I can get some balance. I have done this consciously to feel good and I work with it consciously. I have history of difficult things happening in life; therefore, I feel proud that I have managed it – now I go and sing and make sure that I get out and away…dance and try to do the things that I'm good at and that's fun, to get some air under my wings. I've learned and I feel proud over this.* (Informant)

Overall, the analysis shows that parents' strategies for organizing their lives in regard to their informal care responsibility can be stated as strategies that make life worth living by providing room for own growth and development.

However, there are also negative* circumstances such as losses in life* related to the care responsibility of the ACWS, leading to the project and strategies to control life. These losses make life difficult to live or restrict the parents' actions. Such circumstances may be different kinds of losses in lives. One such unplanned life event was a break-up of a marriage, a life event that also had influenced parents not to remarry.

Other life circumstances related to the care responsibility are damaged relationships within the family or gnawing feelings about responsibility for the family and children. Feelings of powerlessness arose when the parents were unable to protect and support their children in different ways, as described below:

*When xx (her daughter) got ill, my husband became very hateful toward me and also put the hatred on my children. I've never felt so hated; it was like his aggressions went over to my daughter and she kind of threw darts at me. I've never felt so bad, it felt horrible and unfair.* (Informant)

Negative life circumstances were also that one's own needs as an adult had to be put aside at the expense of the parental role and the responsibility to care for the adult daughter or son, as expressed below:

*Life has become more and more limited with xx sickness. I can't even go abroad to work together with the new boyfriend; then, I live alone for months on end and that is not so fun.* (Informant)

Negative life circumstances are also the difficulties in finding time for activities and limited freedom to use their time for various options. These limitations are caused by making parenting the priority and addressing the adult child's needs with various support measures, rather than parents taking care of their own individual needs. In some cases, the limitations are only related to fatigue. The parents are simply too tired to engage in their own leisure activities. An example of such experience is expressed as follows:

*His illness has also limited my daily freedom of action as well as weekends such as Saturday or Sunday, since half of the week xx and I eat dinner together. My social life is also limited because I get fewer free nights off to meet people.* (Informant)

As shown from the analysis, the main point here is that the care responsibility as informal carers is* having control over* their own life as well as over the child's life. Parents describe how they use strategies (parental project, works project, and recreational project) through different assessments that supported their goals and aspirations to be a good parent. They look for ways to parent and rely on relationships within and outside the family to find support for their parenting and care responsibility.

### 4.3. Adaption to the Caring Responsibility

The analysis shows that parents express feelings of great* uncertainty facing all the unknowns* that they encounter in their caring capacity as parent of an ACWS. This uncertainty leads to personal and social adaptions on different levels in order to be able to care. The parents describe how they lack the ability to be able to assess the decisions they make and feel uncertain about them. They do not know how they should act in relation to the children, family, friends, and formal psychiatric care providers. Their children behave abnormally and they do not know how to handle such a situation. The parents describe feelings of fear and fright when they were told that their daughter or son had been given a psychiatric diagnosis. The parents also describe feelings of great frustration when they did not get answers to their questions from the formal psychiatric care services or when they got fuzzy information regarding the proposed care or failed care. Lack of communication between the parents and the formal professional caregivers created many situations that contributed to the parents' insecurities. Such restricting aspects of experiences are exemplified as follows:

*Nobody saw me, nobody told me anything; instead, it was only a shock to see all the people that were constantly sick, someone who just screamed right out and someone who just walked around like a zombie, and to know that there was my son. I thought that he would always be there and that he'd always be so crazy… “what a scream” the painting by Munch of the anxiety cry (scream). As a whole, I was like that painting. *(Informant)

However, the analysis shows also that the adaption to a life with caring responsibility can be supported by* positive events that made life worth living* and that developed the parents' own self-image.

The analysis shows how valuable it is to be met with positive attitudes from others, for the family to feel good, or to be able to see the child develop positively. The parents felt strengthened by the reaffirming social relationships and by increasing their own knowledge.

The parents in this study expressed in many different ways that they became positively involved in their caring responsibility when someone points out the merits of their children—for example, when the formal healthcare staff speaks positively about the son or daughter. This can be praise for the ability to cope with different things or expressing that the children are well liked within the staff group of service providers. All these aspects of social confirmation support the adaption to life with a caring responsibility.

It is also important for the parents' own well-being that the family, son, or daughter is doing well, that his/her condition is more stable, or that one can see a positive ongoing development. The parents say they are influenced by how different healthcare interventions work. When the situation is experienced as more stable, the parents feel safe in their adaption to the caring responsibility. Those who had experienced stabilization and perceived that their son or daughter was doing better expressed the impact that this had on themselves as follows:

*I am so happy that he has been so good at taking his medicine and that he has insight into his illness.* (Informant)

The parents also talked about how their own self-image was influenced by their* perception of health and activity*. They stressed the importance of a good relationship with their child and the experience of working in a positive parental role.

Important for the adaption to the caring responsibility was repairing the relationship when the parents had accused each other of being at fault for the child's illness. Parents describe how stressful feelings of guilt had caused them to realize that mental illness can affect people just like any other disease and that one cannot find blame. Parents also expressed how the child's illness contributed to their own greater self-knowledge and strength and pride in being able to cope. Such positive self-image, personal growth, and dignity are described as follows:

*I've gotten a whole new dimension in life, you could say, through a new way to see things about life and people. So after all, the disease has given me positive signals. *(Informant)

The formal healthcare provision was viewed often as supportive in the parents' informal care responsibility, when the healthcare workers provided the care, or when a meeting with a healthcare representative went well. This is expressed as follows:

*I feel that the healthcare has been good for xx. He has gotten a lot of good support from hospitals, rehabilitation, the employment service, and the social insurance company. I give [them] 100 points. But support for family members, nothing exists.* (Informant)

Parents also give an account of* negative* aspects of the caring responsibility including* circumstances that made life difficult to live*. The analysis shows that the parents were strongly affected by the negative social attitudes directed toward the mentally ill in the community. Their own physical and mental health was, as the parents experienced it, affected by those attitudes. Parents described that some relationships with other people are difficult to manage. They even accused themselves of not being good enough as parents. Due to the accusations, the parents felt that they were the cause of their child's disease or that they were bad parents. Some parents were reluctant to talk about their child's illness with their relatives and friends. The son or daughter's diagnosis of schizophrenia was perceived as a negative stigma, and parents also felt relief when the child had not yet been diagnosed. Feelings of being questioned in their parental role by the health professionals were experienced as a lack of dignity, described as follows:

*This diagnosis that he has gotten is extremely frightening. No one in society knows that there are subgroups of different types of psychoses. All these people don't chop someone with an axe or put a knife into people; not all of these people do that.* (Informant)

In regard to parents' adaption to the informal care responsibility, the analysis showed that the more strategies and goals the parents had in their care responsibility, the more they described their own health status as being better. Thinking that life was still worth living, feeling fine themselves, and being in good health were interpreted as indications of how the adult child was doing.

Parents described their physical and mental health as being good and then immediately supplemented this data with descriptions of both physical and psychological symptoms of ill health. Descriptions of illness that parents had experienced included, for example, obesity, insomnia, back pain, increased blood pressure, effects on the cardiovascular system, muscle inflammation, vocal cord inflammation, and breathing difficulties. The parents linked all of these symptoms to the fact that they were responsible as carers and that their son or daughter was mentally ill. These difficulties of the relationship were experienced as follows:

*So, I've plugged up my ears shut so I won't hear so much, because otherwise my head starts to freeze. I get a headache and then I start to get nauseous, so physical stuff, so I'm in real bad shape…I've been extremely strong physically, but now it's hard to mow the lawn, I get injuries, so I have to think about what I'm doing. There are strains, inflammations. Really strange stuff. One can't believe it's true. The whole thing is because I can't get any deep sleep; I wake up every fifteen minutes. It has been like that for so long that the body has started to take a beating. First, came the lack of deep sleep, then came the problem with the muscles, and thereafter, came the problems to tolerate sounds. *(Informant)

As shown, when the responsibility for care in daily life becomes too large, it is handled by switching off perceptions or by experiencing illness. In regard to experiencing mental illness, the parents described feelings of anxiety and uncertainty of what might happen with the adult child: if the son or the daughter would take their own life or set fire to their house. The parents described the anxiety that affects them and the insecurity they feel in terms of the help they received and did not receive as follows:

*Now I can't handle it anymore, when I feel that stressed then I'm ready to/…/so I think that now I have to go in to the psychiatric emergency department.* (Informant)

The experience of health problems was related to the parents' responsibility as carers and was often linked to the social situation as a whole. Some parents were divorced, and the relationship with the former husband/wife had not been the best, which also affected sibling relationships negatively. The guilt feelings were palpable when the parents blamed themselves for the bad relations within the family as well as for the external relations toward the nursing staff.

The fear of becoming sick themselves existed, but the most critical threats to the parents' health as was experienced were the feelings of powerlessness and anger about not being good enough, not being able to protect and help one's own child, and not being able to access healthcare or get sufficient healthcare.

The parents' own health was also adversely affected by the negative attitudes of the formal nursing staff toward the parents, as the parents experienced it. This, in turn, increased their own feelings of guilt when it came to their inadequacies in the caring responsibility, their parenting role, and contact with others, which is expressed by a parent as follows:

*Well, everything is pretty week…very up and down, I don't feel a part of life, I have taken a step aside, I don't have the strength to be with people, I have stepped aside.* (Informant)

Without professional healthcare support in the caring role, the social network has an even greater importance. The parents are critically of limited access to formal healthcare as well as limited choices of healthcare resources. They mentioned that a lack of trust in healthcare occurs when being unable to understand the behavior of nursing staff, the information provided, or the prescribed drug treatment. The lack of trust was expressed as follows:

*That doctor that tells my son when he asks him about the medication, “well, if this doesn't help you, what should I do then?” (Nozinan, is that what that fucking rat poison is called)… “Well, then you'll have to pray to God,” answers the doctor. I mean, how can someone answer in that way.* (Informant)

As the analysis shows, the main point here is that the adaption to the caring responsibility and to being the parent of an ACWS is filled with uncertainty and is a process mixture of struggling, cooperation, and compromise. However, adaption is believed to contain an element of being involved in a social relationship with others. The parents felt strengthened by such involvement.

## 5. Discussion

The contributions of this paper will be discussed in relation to substantive, theoretical, methodological, and practical aspects.

*Substantively*, this analysis contributes to understanding the meaning that parents provide about their informal care responsibility. The expressed goal is to be a good, responsible, and accountable parent who cares not only about their ACWS, but also about the whole family. Furthermore, the study contributes to understanding how parents construct and manage their caring responsibility by adaption to it in a process that combines struggle, cooperation, and compromise, not only* outwardly* toward the social context in which they find themselves, but also* inwardly* toward the family and themselves.

So, what constitutes being a good parent and what are the consequences?* Inwardly*, the moral foundation of altruism and love as well as the rational consideration of their child is a crucial explanatory aspect when studying how parents understand being a good parent [12, 29]. This also explains why parents fight to get the child's different needs met and to get support for this care responsibility from the formal psychiatric healthcare system. Another perspective appears when one studies parents' personal motive of being a good parent. That means improving their readiness for parenting by searching for knowledge and understanding of mental illness and health. This readiness also involves parents' own growth and development. Parents need support and knowledge about mental illness. However, that support is not adequate if one looks at the formal psychiatric care support available for relatives who are taking care of individuals with a mental illness. The Interest Association for Schizophrenia (IFS) is of high importance in regard to supporting parents' readiness to care. We assert that support from the IFS is crucial not only for giving psychological and social support, but also to educate and serve as a knowledge center for parents and patients.

*Outwardly*, being a good parent living with the caring responsibility for everyday lives means to construct and perform the care by constructing an identity as a good responsible parent and by practicing accountability. In other words, the informal care responsibility is understood as being an accountable, good parent who also performs the accountability to the surrounding community, especially towards formal healthcare professionals. This kind of identity construction, of good and responsible parenthood in terms of altruism, love, and consideration, seems in terms of values to be faced as a moral self-identity in presentation of oneself [40, p. 9 and part II]. Gecas [41] supports these findings by emphasizing that identity has become a fertile ground for understanding personal experiences and the relationship between self and society. Values constitute an important location for identity construction, and “the self-conception starts with values and aspirations, and continues to be represented in value and aspiration terms.” That is, values give meaning, purpose, and direction to our lives (ibid.).

However, this self-presentation is located in the social and cultural context and structural space between the informal and formal care service positions. The informal care position as parent, family member, and member of the Interest Association for Schizophrenia (IFS) deals with shared self-definition. This seems to be an important source of identity and of care rationality in the interaction with care professionals within the formal psychiatric care. Often, motivation to be a good parent also involves implicit expectations to fulfill the ascribed roles of a parent. However, is such self-identity and rationality presentation perceived and considered by the formal healthcare professionals? The question to be asked is, when it manifests, is extensive supportive and confirmatory care given to the parents? In this study, it seems that this interaction between parents and healthcare professionals is uneven, working very well in some cases and very poorly in others. However, there is still need of knowledge in this issue about whether these experiences differ from the fathers' versus the mothers' perspective.

A common feature of the meaning-making of the care responsibility as it is experienced is the centrality of both protective parenthood and accountable self-performance as a carer. The caring responsibility is understood as a link between the child, family, and the outside world. In this informal position, the parents' ability as an active parent with good social relations to family members and with the psychiatric care professionals is crucial.

The parents organize and use strategies to make sense of and endow their care responsibility by giving meaning within the context of their personal and social relationships and employment. The strategies used are parental projects, work related projects, and recreational projects. These projects are ways to control the life situation when feeling out of control. The key finding here is that the more projects the parents had, the more they seemed to experience well-being. This highlights the importance of considering parents' perspectives and subjective wishes and needs when formal psychiatric care professionals plan support for parents' informal care responsibilities.

Another important form of support from the formal care community is to acknowledge the relatives for the care they provide. It is also clear from findings presented here that life situations for parents are characterized by great uncertainty and, therefore, the parents find it valuable to encounter positive attitudes from others. However, there were many bitter complaints over how society responds to parents' caring responsibility, and the formal care representatives were indicated as approaching the parents with negative attitudes and with limited engagement. Previous studies support these findings [12, 14, 42–44]. According to Pejlert [43], the healthcare system's long patronizing history affects the health professionals' views on the relatives, which can create problems in the contacts between the relatives and the professionals. The relatives can be regarded as being unsuitable as carers for their sick children or healthcare professionals blame the family as a reason for the mental illness, based on theories developed by a group of influential American family therapists in the 1950s. Pejlert [43] writes that the view of the family as a cause of mental illness contributes to keeping the family outside the formal healthcare organization. As shown, the psychiatric care community's knowledge about care, illness, and disease is constructed and developed by claims-makers and interested parties. Therefore, a number of policy implications can be derived from the key findings concerning the social organization of the parents' world in their care responsibility for their ACWS.

*Theoretically*, the parents' meaning-making of their informal care responsibility and being a good parent not only is based on individual experiences of the parents' informal care responsibility, but is also embedded in the social and cultural meanings of the formal psychiatric care community. As shown, the parents' meaning-making of their care responsibility is also a communicative discursive negotiation with the psychiatric care community and the care professionals about accountability. This involves confirmation of the parents' self-definition and social interaction as responsible parents. This kind of confirmatory care or lack of the same can have long-term consequences for parents' health and well-being. One key finding from this study is that parents experience their caring responsibility with a great uncertainty which also gives them feelings of physical illness such as back pain, increased blood pressure, effects on the cardiovascular system, muscle inflammation, vocal cord inflammation, and breathing difficulties.

The parents' desire for information and knowledge is at stake in the discursive negotiations with the healthcare professionals. This study shows that parents have a great need to try to understand the illnesses and, above all, the knowledge that will enable them to provide appropriate support for their child. Instead of formal confirmatory care (e.g., information, social, emotional, and practical support), the parents expressed both frustration and anger over the construction of rigid rules and laws. They found themselves facing roadblocks. However, the information that was supportive could encourage the parents to regain or hold on to the social networks and thereby promote the informal care responsibility for the ACWS. Previous studies support these findings [10, 44–46].

The contribution of knowledge here is an insight into the complexity of parents' informal care responsibility as well as how its meaning is interactively produced. This is a key factor in the establishment of a functioning confirmation of care in order to support services to parents as informal carers. This knowledge contribution is also important to our understanding of the informal care position in mental health and illness in society. This theoretical perspective has guided us in suggesting political implementation of future informal care responsibility.

When it comes to new theoretical understanding based on this study, the parent's meaning-making involves a tension between care and concern for their own interests on the one hand and care and concern for the child's interests on the other. Being able to be a good parent and to handle the caring responsibility, the parent must compromise. In this tense situation, parents compromise between elements of struggle, cooperation, avoidance, and adaption in their interaction with the world outside as well as the world inside.

This approach can make a significant contribution to our understanding of the social and experiential dimensions of caring responsibility and it can help us broaden policy deliberations and decisions.

*Methodologically*, this paper reflects the special contribution that can be made by qualitative analysis to understand and uncover the micro and macro processes in psychiatric care and the relationship of formal care professionals with informal carers, here, parents. In other words, by giving voice to the parents' perspective, the experience of their care responsibility approach can lead to important clinical reforms and to changes in how society responds to related parties who are informal carers.

The perspective analysis shows that language use in the dialogues between the informants (the parents) and the interpretative analysts (the researchers) bridges the gap between the parents' meaning-making and the factual situation of being the informal carer. The question of validity implies that the interpretations in the study are checked against different criteria [47]. In this study, the main criterion is a valid interpretation that gives meaning to the phenomenon being studied, here the parents' informal care responsibility. In the chosen method, context, analysis, and interpretation have been made transparent so that the reader can follow and understand how these relate to each other.

When it comes to the limitations of this study, it can be concluded that the reported care responsibility experiences may not be reflective of carers in other settings because the sample used in this study was purposive. However, the main interpretation about parents' experiences as informal carers as being a good parent and their compromise between struggling, cooperation, avoidance, and adaption to the world outside as well as the world inside can be extended beyond its immediate context. This in turn increases the ability to apply it outside the study's context.

*For practical considerations*, however, these findings are of more than academic interest. They also point to significant psychiatric care policy from the perspective of confirmatory care, which are understood as care on a personal level. It is crucial for the healthcare professionals to be aware of the perspective that reflects the parent's perspective on informal care responsibility to be able to support care service to the parents. Similar findings were developed by Siouta et al. [48–51] and the Swedish Society of Nursing [52]. Creating an active role for the relatives requires knowledge based on the parents' preferences [53, 54]. This will give the healthcare providers a better understanding and willingness to cooperate with the relatives [48, 49, 54].

To that end, there is a need for an in-depth debate about formal psychiatric care's responsibility to support the relatives who are informal carers of psychiatric patients. It is most important to establish a policy for how the psychiatric care is to be divided between the formal and informal care givers.

There is a lack of research in the area investigated in this paper, and a number of issues regarding the informal care responsibility are still unexplored. One such area is in relation to what experiences the healthcare professionals have with parents who expect support from the mental healthcare system and examining how professionals support parents.

*The Main Conclusions and Practical Considerations*

- It is crucial for healthcare professionals to be aware of parents' perspectives as being responsible for informal care so as to be able to support a care service for parents.
- There is a need for an in-depth debate about professional psychiatric care's responsibility to support the relatives who are informal carers of psychiatric patients.
- It is most important to establish a policy for how psychiatric care is to be divided between the formal and informal care givers.

## Figures and Tables

**Table 1:** Characteristics of parents interviewed (*N* = 8).

Sex	Age	Child sex and age	Work time	Sickness degree	Retirement
Female	58	Son, 27	100%		
Female	58	Daughter, 25	100%		
Female	56	Son, 30	50%	50%	
Female	52	Daughter, 23	80%	20%	
Female	57	Son, 22		100%	
Male	63	Son, 31	80%		20%
Male	59	Son, 30		100%	
Male	59	Son, 27	100%		

**Table 2:** The finding presented as theme, categories, and subcategories (theme: the meaning of being good, responsible, and accountable).

Categories	Subcategories
The social organization of the parent's world as informal carers in form of strategy projects	*Parental projects *
	*Work related projects *
	*Recreational projects *
	*Repertoires of controlling the life situation and feelings of being out of control *

Adaption to the caring responsibility	*Uncertainty facing all the unknowns*
	*Positive events that made life worth living*
	*Perception of health and activity*
	*Negative circumstances that made life difficult to live*

## Appendix

See Tables 1 and 2.
